# GRP78 in lung cancer

**DOI:** 10.1186/s12967-021-02786-6

**Published:** 2021-03-21

**Authors:** Shengkai Xia, Wenzhe Duan, Wenwen Liu, Xinri Zhang, Qi Wang

**Affiliations:** 1 Department of Respiratory Medicine, The Second Hospital, Dalian Medical University, No. 467 Zhongshan Road, Dalian, 116023 China; 2 Cancer Translational Medicine Research Center, The Second Hospital, Dalian Medical University, Dalian, 116023 China; 3 Department of Respiratory and Critical Care Medicine, The First Hospital, Shanxi Medical University, No. 85 Jiefang South Road, Taiyuan, 030001 Shanxi China

**Keywords:** GRP78, Lung cancer, Endoplasmic reticulum, Unfolded protein response (UPR), Autophagy

## Abstract

Glucose-regulating protein 78 (GRP78) is a molecular chaperone in the endoplasmic reticulum (ER) that promotes folding and assembly of proteins, controls the quality of proteins, and regulates ER stress signaling through Ca^2+^ binding to the ER. In tumors, GRP78 is often upregulated, acting as a central stress sensor that senses and adapts to changes in the tumor microenvironment, mediating ER stress of cancer cells under various stimulations of the microenvironment to trigger the folding protein response. Increasing evidence has shown that GRP78 is closely associated with the progression and poor prognosis of lung cancer, and plays an important role in the treatment of lung cancer. Herein, we reviewed for the first time the functions and mechanisms of GRP78 in the pathological processes of lung cancer, including tumorigenesis, apoptosis, autophagy, progression, and drug resistance, giving a comprehensive understanding of the function of GRP78 in lung cancer. In addition, we also discussed the potential role of GRP78 as a prognostic biomarker and therapeutic target for lung cancer, which is conducive to improving the assessment of lung cancer and the development of new therapeutic interventions.

## Introduction

Lung cancer is the leading cause of cancer-related mortality in men and the second most diagnosed malignancy in women, only after breast cancer [1]. In 2018, lung cancer accounted for more than 1.8 million deaths worldwide [2], with an overall 5-year survival rate of approximately 19% [3]. The survival rates of lung cancer patients are largely dependent on the early diagnosis and intervention of the disease. An improved understanding of the molecular mechanisms involved in the development and progression of lung cancer, along with the development of drug resistance, will aid in the discovery of new treatment strategies.

Glucose-regulated protein 78 (GRP78), also known as immunoglobulin heavy chain binding protein (BiP), together with GRP94 and GRP58, was discovered as a cellular protein inducible by glucose starvation in the late 1970s [4]. GRP78 has a signal peptide sequence that acts as a molecular chaperone on the endoplasmic reticulum (ER). It is involved in the proper folding and assembly of proteins, proteasome degradation of misfolded proteins, ER and Ca^2+^ binding, and the activation of transmembrane ER stress sensors [5]. Under ER stress, activated GRP78-related unfolded protein response (UPR) restores cell homeostasis or induces cell death in chronically damaged cells [6]. As such, GRP78 is overexpressed in lung cancer [7, 8], and is widely involved in the promotion of tumor proliferation, metastasis, drug resistance, and apoptosis [9, 10].

In recent years, new insights into the chronic ER stress experienced by cancer cells, along with the importance of GRP78 or GRP78-related UPR in tumor progression, have stimulated the exploration of GRP78 and UPR as potential therapeutic targets. In this review, we describe the role of GRP78 in the tumorigenesis, apoptosis, autophagy, invasion, and metastasis, and drug resistance of lung cancer, as well as its clinical implications as a potential prognostic and therapeutic target of this disease.

## GRP78 in UPR and stress response

**Fig. 1:**
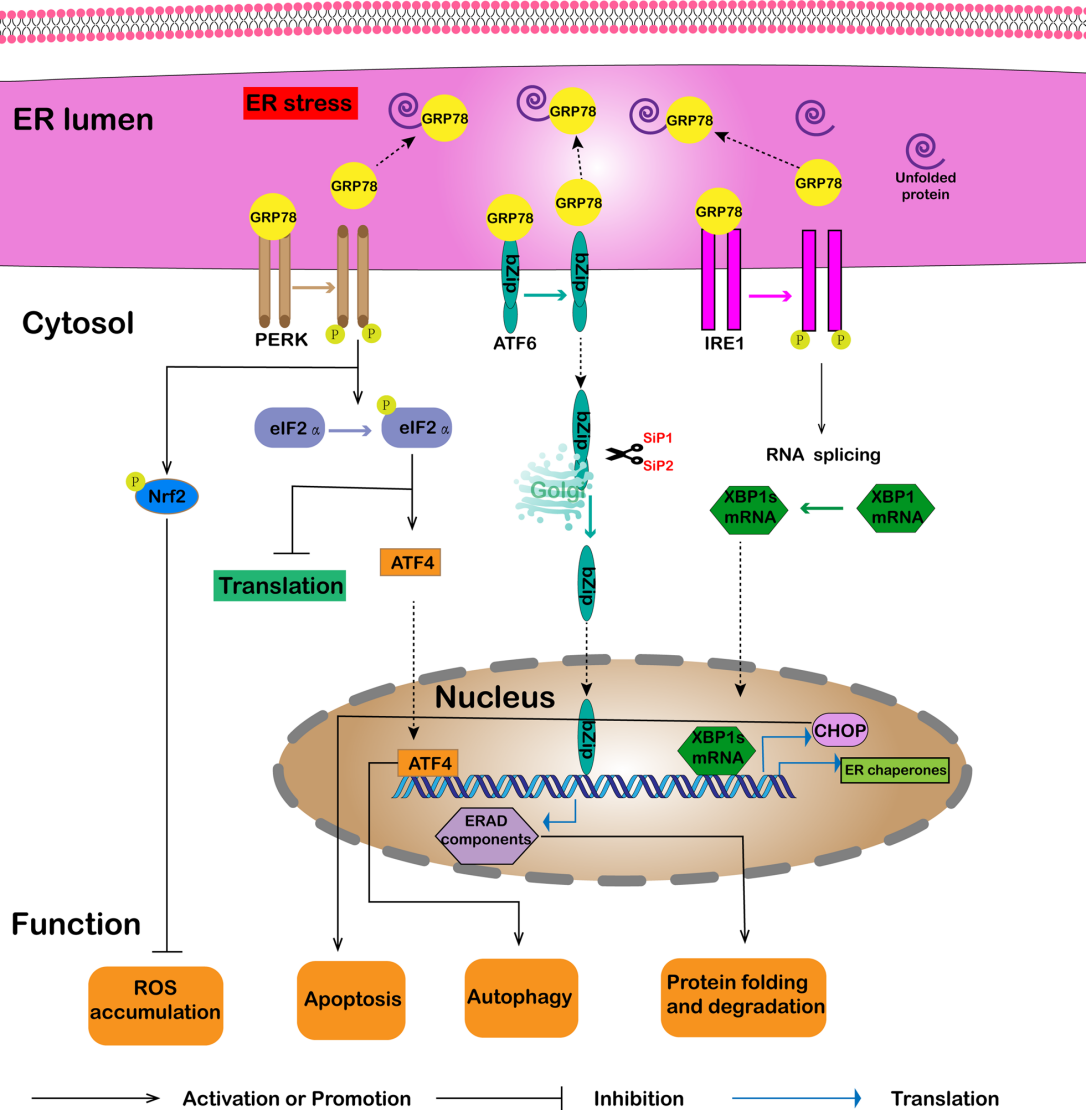
GRP78 in UPR and stress response. During ER stress, when misfolded proteins accumulate in the ER, GRP78 binds to the misfolded proteins, keeping them foldable and releasing three ER transmembrane proteins. The three proteins (IRE1, PERK, and ATF6) are activated to initiate the UPR signaling cascades. Activated IRE1 cleaves unspliced XBP1 mRNA, and the cleaved mRNA is then translated into the transcription factor XBP1s. PERK dimerizes, trans-autophosphorylates, and then phosphorylates eIF2α to inhibit protein translation. Phosphorylated eIF2α also activates the transcription factor, ATF4. ATF6 is transferred from the ER to the Golgi complex. ATF6 is cleaved in the Golgi by SiP1 and SiP2 proteases, releasing a cytosolic basic leucine zipper (bZIP) domain, which is transferred to the nucleus. Three transcription factors, including ATF4, XBP1s, and ATF6, synergistically control the expression of adaptive genes related to ERAD, ER-chaperones, pro-apoptotic CHOP, and autophagy, to maintain steady-state. In addition, activated PERK phosphorylates NRF2, thereby generating an antioxidant response to inhibit ROS accumulation

The ER is a perinuclear organelle, involved in the synthesis and folding of secreted nuclear and membrane proteins. These proteins are then transported to the cell surface through the cross-Golgi network [11]. However, the function of the ER can be hindered by several factors, such as hypoxia, nutrient/glucose deficiency, oxidative stress, Ca^2+^ depletion and certain viral infections [12]. ER stress is induced by alterations in its folding abilities, resulting in an imbalance of protein homeostasis. In return, the proteins become misfolded, which has detrimental effects on their normal cellular function. To avert this situation and re-establish homeostasis, the cell triggers the UPR to activate several biochemical mechanisms and alleviate the ER stress. In situations where the UPR cannot re-establish homeostasis, cellular apoptotic pathways are activated (Fig. 1) [12–14].

IRE1 (inositol-requiring enzyme 1), PERK (PRKR-like ER kinase), and ATF6 (activating transcription factor 6) are the three major ER transmembrane proteins involved in the UPR signaling cascade. GRP78, which binds these three proteins in ER through their lumen domains, is considered to be the main regulatory protein of UPR [4]. In the normal state, GRP78 mainly binds to IRE1, PERK, and ATF6 to silence these proteins to prevent UPR signaling. However, GRP78 combines with the misfolded proteins accumulated in the ER under stress conditions to keep the proteins in foldable states and releases three UPR mediators. Next, it releases the three UPR mediators, each of which induces a different signal transduction pathway that mediates the UPR of a particular arm.

IRE1 can dimerize and undergo autophosphorylation when GRP78 releases IRE1, thereby activating its ribonuclease (RNase) [15, 16]. Next, the mRNA of XBP1 (x-box binding protein) is cleaved, which generates a spliced form of XBP1 (XBP1-s) [17]. XBP-1s upregulates genes that encode ER chaperones and ER-related protein degradation (ERAD) proteins. In addition, it mediates the transcription of XBP-1 in a self-regulated manner [18]. Next, IRE1 activates c-jun n-terminal kinase (JNK) by recruiting tumor necrosis factor (TNF) receptor-related factor 2 (TRAF2), which promotes apoptosis under the extended UPR signaling pathway [19].

PERK acts similarly to IRE1 after the release of GRP78, leading to the dimerization and phosphorylation of the eIF2 promoter (eukaryotic translation initiation factor 2a), which inhibits global protein synthesis [16]. The phosphorylation of eIF2a activates ATF4 (activating transcription factor 4), which controls the expression of UPR target genes related to ERAD, protein folding, amino acid biosynthesis, autophagy, and pro-apoptotic CCAAT/enhancer-binding protein-homologous protein (CHOP) to regulate the UPR [20–23]. CHOP is a characteristic factor of ER stress that causes cellular apoptosis [24]. Besides, PERK inhibits ROS accumulation by phosphorylating and stabilizing NRF2, which regulates the expression of genes containing antioxidant response [25].

After GRP78 is release, ATF6 is transferred to the Golgi complex, where ATF6 is cleaved by the SiP1 (site-1-protease) and SiP2 (site-2-protease) proteases, which releases a cytosolic basic leucine zipper (bZIP) domain [26, 27]. The bZIP domain translocates to the nucleus, promoting the transcription of ERAD genes and XBP1 [28], which further reduces the burden of unfolded proteins and ER stress.

## Biological function of GRP78 in lung cancer

**Fig. 2:**
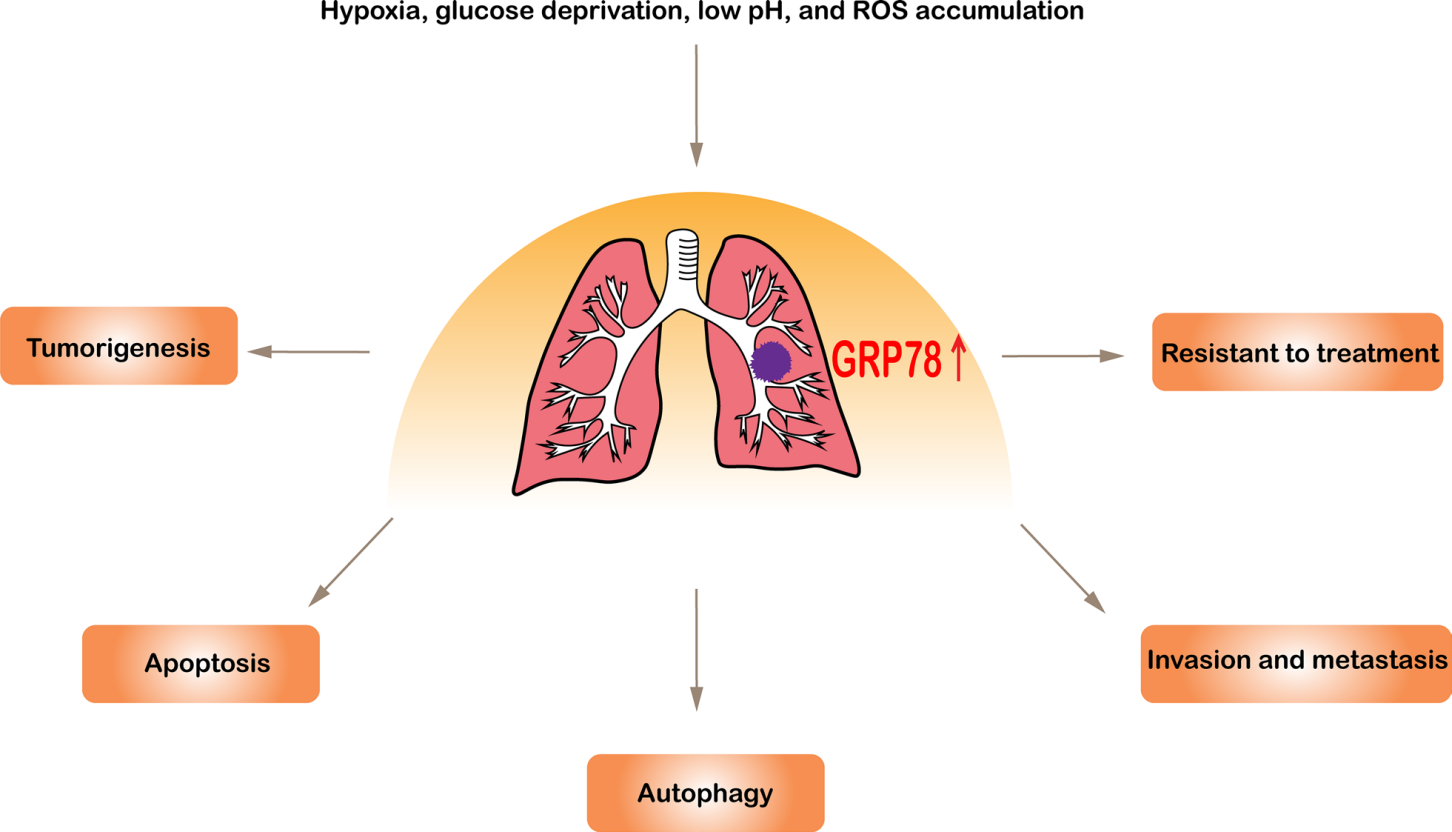
Role of GRP78 in lung cancer. Complex conditions in the tumor microenvironment lead to the upregulation of GRP78 and its cell surface expression in lung cancer. GRP78 regulates lung cancer tumorigenesis, apoptosis, autophagy, invasion and metastasis, and chemotherapy resistance in a UPR-dependent and UPR-independent manner (see text for more details)

UPR is activated to support tumor survival and development due to the poor tumor vascular hyperplasia and high proliferation, making tumors suffer from various forms of stress. As the main UPR regulatory protein, GRP78 is highly expressed in a variety of tumor cells. In this section, GRP78 has both UPR-dependent and UPR-independent functions, which may help in several aspects of tumor biology, including tumorigenesis, apoptosis, autophagy, invasion, and metastasis, along with chemotherapy resistance (Fig. 2).

### Role of GRP78 in tumorigenesis

In the early stages of tumorigenesis, ER stress increases the expression of GRP78 and other ER chaperone proteins. Compared with normal lung tissues, GRP78 is significantly overexpressed at the mRNA and protein levels of lung cancer tissues. The overexpression of GRP78 in lung cancer tissues is closely related to the differentiation and development of lung cancer [7, 29].

On the one hand, oxidative stress may have profound effects on the function of the ER by interfering with various aspects of protein folding, leading to the induction of the ER stress response [30], but the enhanced activity of GRP78 can relieve oxidative stress, which plays a vital role in protecting cancer cells from oxidative stress-induced cellular damage and cell death [31, 32]. The overexpression of GRP78 induced by this continuous oxidative stress can help cells become more resistant to various stressful challenges, which contributes to the development of tumors. For example, during the entire course of nicotine exposure, nicotine causes an acute increase in reactive oxygen species (ROS), which induces GRP78 upregulation and subsequent PERK phosphorylation, causing ER stress and UPR activation. In return, this produces a favorable microenvironment suitable for the development of lung cancer [33]. In microfluidic devices, continuous low flow cigarette exposure to non-tumor bronchial epithelial cells in chronic obstructive pulmonary disease (COPD) and lung squamous cell carcinoma patients stimulates ROS production and induces tumor-like transformation of human non-tumor bronchial epithelial cells through GRP78, NF-κB, and PI3K signaling pathways [34].

On the other hand, GRP78 enhances the folding ability of ER protein and protects cells from ER stress-induced cell damage and cell death, which has many effects on tumorigenesis. RRBP1 enhances GRP78 protein expression and regulates UPR, which allows it to adapt to ER stress to maintain the tumorigenicity of lung cancer [35]. OTUD3 plays an oncogenic role in lung cancer cells and promotes the development of lung cancer by directly interacting with GRP78, where OTUD3 deubiquitylates GRP78 and maintains GRP78 stability in vitro and in vivo. An important note is that OTUD3 co-localizes with GRP78 in the cytoplasm, especially in the ER, indicating that it causes carcinogenesis through the ER [36]. These mechanisms suggest that the regulation of GRP78 can impact the degree of ER stress and oxidative stress, which may play a role in the development of lung cancer.

### Apoptosis

**Fig. 3:**
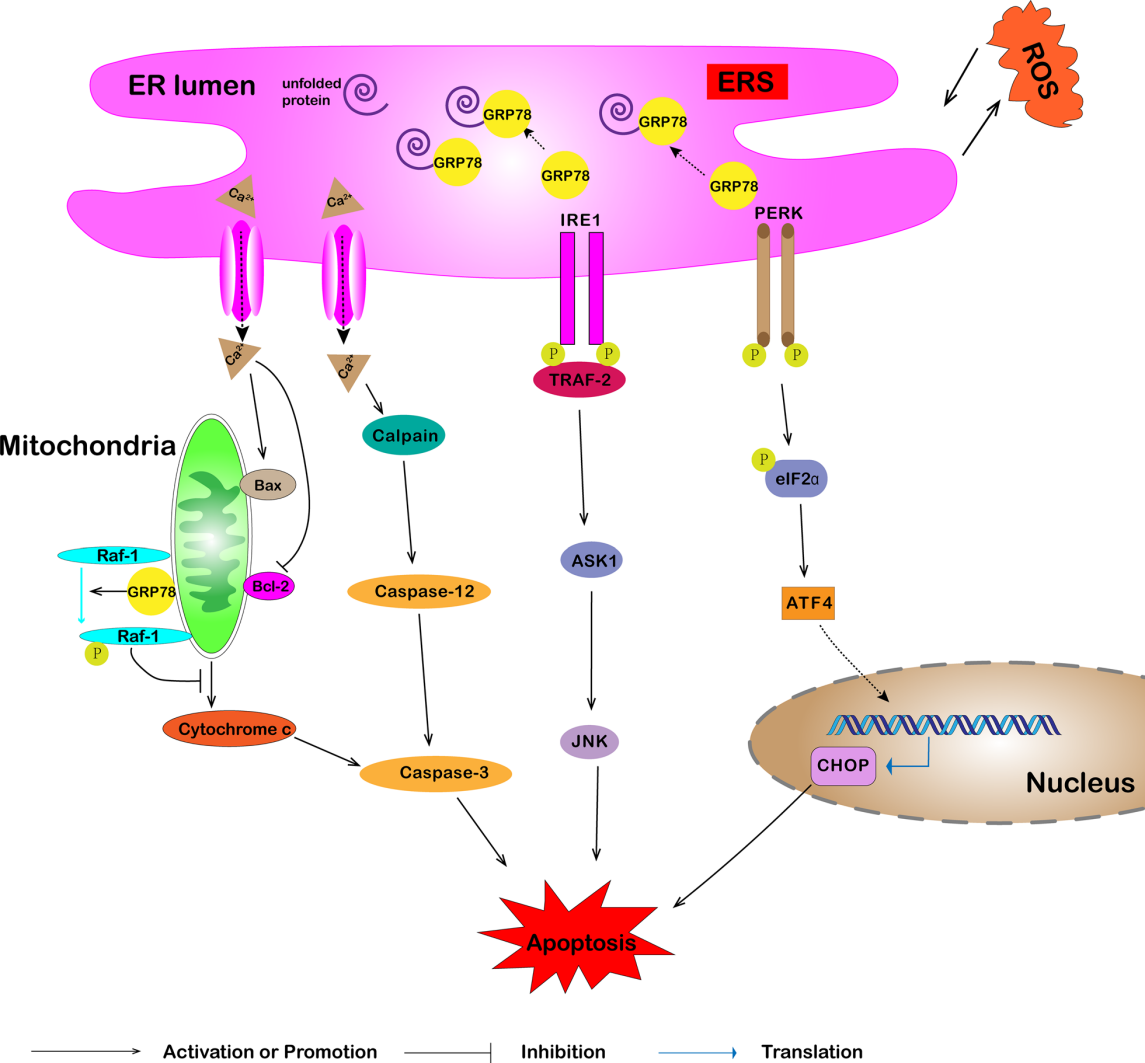
Role of GRP78 in cellular apoptosis. In the case of strong and persistent ER stress, GRP78 is upregulated, which promotes the production and release of Ca^2+^. GRP78 activates UPR signaling to upregulate CHOP and induce cellular apoptosis. Increased [Ca^2+^] induces apoptosis in a mitochondrial-dependent pathway by increasing Bax and decreasing Bcl-2 expression. In addition, Ca^2+^ released into the cytoplasm can also activate calpain. Activated calpain promotes the cleavage of caspase-12 and mediates apoptosis through a cascade reaction. JNK is also activated by dimerized IRE1 via TRAF2-ASK1 signaling to induce apoptosis. Persistent ER stress can cause excess ROS production, and high ROS levels can induce ER stress, forming a vicious cycle. GRP78 can reduce the release of mitochondrial cytochrome c to inhibit apoptosis by stabilizing the expression and activity of Raf-1

Apoptosis is an active and physiological death process that occurs under certain physiological or pathological conditions, controlled by inherent genetic mechanisms. In recent years, several studies have assessed the role of GRP78 in cell apoptosis (Fig. 3).

On the one hand, GRP78 has an important anti-apoptotic function, and several cancer cell lines adapt to chronic stress in the tumor microenvironment via GRP78 upregulation [37, 38]. GRP78 can stabilize mitochondrial permeability, inhibit mitochondrial cytochrome c release, and reduce ER stress-induced apoptosis by maintaining the stability and function of Raf-1 [39]. Ectopic expression of GRP78 can form a complex with caspase-7 and caspase-12 that inhibit the activation of caspase-12-mediated cell death [40]. At the same time, kurarinone induces the apoptosis of A549 cells by inhibiting GRP78 expression and subsequently releasing the inhibition of caspase-12 and caspase-7 [41]. On the other hand, some studies have shown that GRP78 expression is positively correlated with the apoptosis of human lung adenocarcinoma cells [42]. The enhanced activity of GRP78 increases the expression of cleaved caspase-12 and BAX, promoting the apoptosis of A549 cells [43]. Ca^2+^ imbalance is an important factor that mediates apoptosis. For example, GADD153 and GRP78 are upregulated in response to ER stress, promoting the production and release of Ca^2+^. Increased [Ca^2+^] induces apoptosis in A549 cells through mitochondrial-dependent pathways, such as upregulated Bax expression and downregulated Bcl-2 expression [44]. In addition, Ca^2+^ released into the cytoplasm can activate calpain, which is transferred to the ER membrane to promote the cleavage of caspase-12, mediating the apoptosis of lung cancer cells [45].

Multiple studies have shown that GRP78-related ER stress signaling pathways are involved in cancer cell death [46]. For example, GRP78 activates CHOP through the PERK pathway [20–22]. CHOP stimulates the transport of Bax to mitochondria and inhibits the expression of Bcl-2, promoting mitochondrial pathway-mediated apoptosis [45]. The excessive activation of IRE1 can induce apoptosis [47]. ER stress increases the expression of GRP78, CHOP, and IRE1α, in which activated IRE1α combines with TRAF2, recruits ASK1 to form a complex, and then activates JNK to induce lung cancer cell apoptosis [48–50]. In addition, continuous ER stress can increase ROS production, and high ROS levels can induce ER stress, forming a vicious cycle that results in cell apoptosis [51, 52]. Hence, GRP78 is the main protein that regulates the apoptosis of lung cancer cells. On the one hand, GRP78 plays an anti-apoptotic role by inhibiting the functions of apoptosis-related proteins. GRP78 also exerts a pro-apoptotic effect by mediating Ca^2+^ imbalances and activating the UPR pathway.

### Autophagy

The process of autophagy begins with the isolation of unnecessary by-products or damaged organelles into autophagosomes. Next, the autophagosomes and lysosomes are fused to form autophagolysosomes, which then degrade the internal cargo to provide the cell with its own metabolic requirements, along with the renewal of specific organelles [53]. Autophagy plays both positive and negative roles in promoting the apoptosis of non-small cell lung cancer (NSCLC) cells. In general, the inhibition of autophagy limits the ability of cells to overcome stress and maintain homeostasis [54]. However, there are also examples of autophagy contributing to cell death in lung cancer [55]. There is increasing evidence that ER stress can induce autophagy activation [56–58]. Some studies of lung cancer have shown that autophagy can promote the growth and progression of tumors [59, 60]. ER stress induces autophagy by activating the PERK-eIF2α-ATF4-CHOP and IRE1-TRAF2-JNK signaling axes of UPR, providing a protective effect against β,β-dimethylacrylshikonin (DMAS)-induced apoptosis of human lung adenocarcinoma cells [23]. GRP78 is crucial for autophagy regulation [61]. For example, GRP78 downregulation can block the formation of autophagosomes induced by ER stress [62]. Under acidic conditions, GRP78 knockdown reduces autophagy activation, inhibiting the autophagy-associated cell repair mechanisms in lung cancer cells [63]. In contrast, the hypochlorous acid (HOCl) named probe ZBM-H targets HOCl in the ER, thereby inhibiting the HOCl-induced Lys 353 oxidation of GRP78 and enhancing GRP78 activity. ZBM-H promotes A549 cell autophagy and inhibits lung cancer cell growth through the GRP78/AMPK/mTOR pathway [43]. GRP78, CHOP, and IRE1 are upregulated, followed by the downregulation of the PI3K/AKT/mTOR signaling pathway, which promotes the autophagy of mutant p53-LC cells [64, 65]. Therefore, the life-promoting and death-promoting functions of autophagy in cancer cells are largely dependent on the cell type and stimulus conditions [66, 67].

### GRP78 promotes the invasion and metastasis of lung cancer

The metastasis of tumor cells is one of the most important features of malignant tumors. Tumor metastasis relies not only on the changes of tumor cells but also on the interaction between the tumor cells and tumor microenvironment. The role of GRP78 in tumor metastasis is becoming more apparent due to recent findings. GRP78 expression is upregulated in metastatic tumor cell lines, including lung cancer cell lines [68–74]. The downregulation of GRP78 effectively inhibits the invasion of tumor cells in vitro and metastasis of transplanted tumors [75, 76]. In the next section, we discuss the relevant molecular mechanisms of GRP78-mediated epithelial-mesenchymal transition (EMT) of cancer cells and the interaction between cancer cells and non-cancer cells in lung cancer metastasis.

#### GRP78 mediates the interactions between lung cancer and tumor microenvironment

**Fig. 4:**
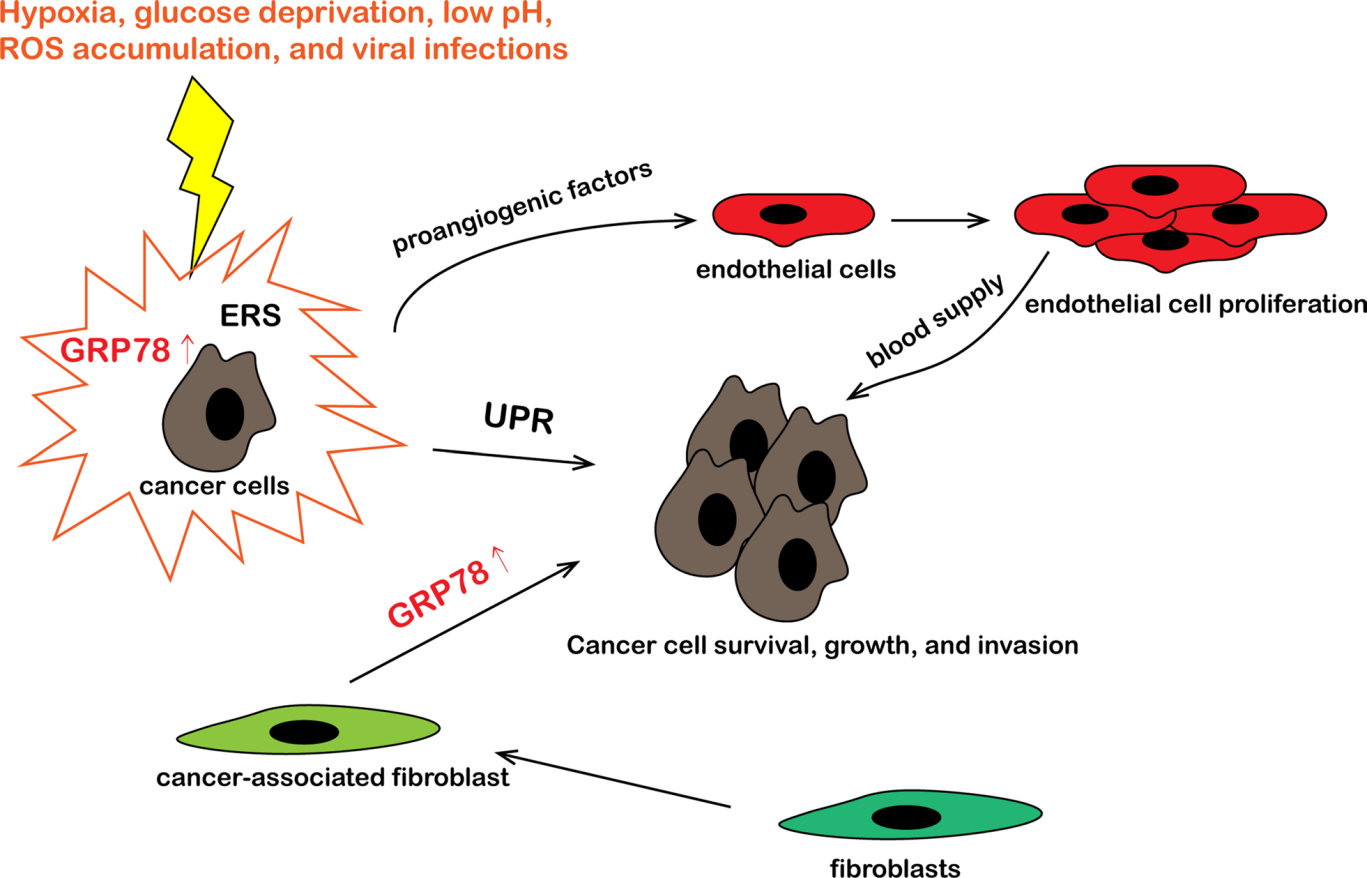
GRP78 mediates the interaction between lung cancer and tumor microenvironment. When the tumor microenvironment is stimulated by hypoxia, glucose deprivation, low pH, ROS accumulation, and viral infections, the cancer cells experience ER stress and increased GRP78 expression. The adaptive UPR is activated to support the survival, growth, and invasion of tumor cells. In addition, the cancer cells secrete pro-angiogenic factors to stimulate the proliferation of endothelial cells, thereby providing nutritional support for the cancer cells. Also, cancer-related fibroblasts promote the invasion of cancer cells by upregulating GRP78 expression

Since solid tumors generally grow faster than the surrounding vasculature, special growth conditions can occur, such as hypoxia, glucose deficiency, and lactic acidosis [77]. Under these stress conditions, the UPR signal is often activated, allowing the microenvironment and tumor cells to adapt to each other. For example, the key ER stress protein BiP/GRP78 interacts with CIM to modulate UPR, enabling lung cancer cells to adapt to the metastatic site microenvironment and acquire the ability to survive and settle at the metastatic site [78]. In addition, non-cancer cells also provide essential support for the growth, angiogenesis, and metastasis of cancer cells (Fig. 4).

Tumor vasculature is critical for tumor growth and metastasis because it provides nutrients and oxygen critical for tumor growth and maintenance. Studies have shown that GRP78 plays an important role in regulating the host vascular system in some tumor microenvironments, including lung cancer [79, 80]. Conditional heterozygotic knockout of GRP78 in host endothelial cells significantly reduced the angiogenesis and growth of tumors, suggesting that GRP78 is an essential mediator of angiogenesis and regulator of cell proliferation, survival, and migration [81]. GRP78-related UPR is also involved in tumor angiogenesis [82]. Hypoxia and glucose deprivation mediate the upregulation of VEGF-A through IRE1 signaling, forming new blood vessels in A549 lung cancer cells [83]. The pro-angiogenic response driven by IRE1 may be secondary to XBP-1 splicing [84].

Cancer-associated fibroblasts (CAFs) play important roles in the occurrence, protection, and metastasis of cancer [85]. CAFs are known to interact with tumor-related immune cells to regulate the microenvironments of tumors [86]. Some studies have shown that GRP78 plays an important role in the differentiation and formation of CAFs, which is conducive to the formation of the tumor microenvironment [87]. In addition, CAFs promote cell invasion in NSCLC cells by upregulating the expression of GRP78 [88].

The role of UPR in the tumor microenvironment suggests that UPR can relieve ER stress at the system level, rather than in a single cellular response. Therefore, it is necessary to understand how UPR, especially GRP78, promotes tumor growth and metastasis by interacting with other cells or microenvironmental components of the tumor. Furthermore, the development of treatments for these effects will improve the therapeutic efficacy and prognosis of patients.

#### GRP78 mediates epithelial-mesenchymal transition (EMT)

Angiogenesis provides a way for tumor cells to escape and metastasize to other organs, but tumor cells must acquire an aggressive and migratory phenotype before they can gain access to the surrounding blood vessels. The abnormal activation of EMT causes tumor cells to lose cell-to-cell contact, resulting in an aggressive and migratory phenotype that is favorable for metastasis. The increase in UPR, especially GRP78, has been shown to promote EMT in various cell types, promoting the tumorigenesis and metastasis of lung cancer. Studies have shown that GRP78 overexpression induces cell–matrix adhesion and EMT by driving TGF-TGF-1-smad2/3 signaling and integrin-1-FAK signaling, which contribute to the metastasis [89]. Under hypoxic conditions, GRP78 expression in A549 cells significantly increases, mediating hypoxia-induced EMT in A549 cells, which is related to the smad2/3 and SRC/MAPK signaling pathways [90]. Polypeptide N-acetylgalactosaminyltransferase-6 (GALNT6) O-glycosylates and stabilizes GRP78, which promotes EMT by enhancing the MEK1/2/ERK1/2 signaling in lung cancer cells [91]. This shows that part of the reason for GRP78′s increased metastatic potential may be its association with EMT.

### GRP78 mediates the resistance of lung cancer to treatment

Patients initially respond to chemotherapy drugs, as demonstrated through reductions in tumor size. However, many patients develop resistance to chemotherapies because a small number of cancer cells adapt internally in response to the additional stress caused by chemotherapeutic drugs. The adaptation to chemotherapeutic drugs may be due to factors like enhanced drug efflux, drug activation/inactivation, alternations in drug targets that promote the removal of drug from the tumor, as well as the intrinsic changes of tumor cells, like enhanced stemness [92]. Here, GRP78 is proved to mediate the resistance of lung cancer cells to treatment through UPR and promoting the stemness of cancer cells.

#### GRP78 mediates lung cancer resistance to treatment through UPR

The adaptability of using UPR is also a mechanism used to ensure the survival of cancer cells after exposure to chemotherapy drugs. In many cell types, excessive GRP78 reduces apoptosis, alleviates ER stress, and increases the chemo- and radio-resistance of tumor cells [93]. The knockout of GRP78, ATF6, ATF4, and XBP1s is related to the mechanism of increasing the sensitivity of cells to chemotherapy drugs [94–96]. Photodynamic therapy (PDT) can upregulate GRP78, which has a cytoprotective effect and makes the cells resistant to PDT [97]. RRBP1 enhances the expression GRP78 to make lung cancer cells resistant to chlamycin, 2-deoxyglucose, and doxorubicin [35]. GRP78 expression is upregulated on the cell surface, which regulates AKT phosphorylation. GRP78 may enable ER stress tolerance (ERST) in lung cancer cell lines to survive more easily under cisplatin treatment than parental cell lines by activating the AKT signaling cascade [98].

#### GRP78 mediates the resistance of lung cancer to treatment by acquiring stemness

Cancer stem cells may have many characteristics of normal stem cells, including being relatively quiescent, resistance to drugs and toxins by expressing drug-resistant molecules, active DNA repair capabilities, and resistance to apoptosis. These properties allow cells to survive chemotherapy and repopulate the tumor [99]. Recent studies have shown that GRP78 on the cell surface (sGRP78) transmits signals to promote epithelial-mesenchymal transition and stemness of cancer cells [31, 91, 100]. Compared with other populations in tumors, sGRP78^+^ cells express higher levels of stem cell genes [101]. In addition, the increase in GRP78 value in gefitinib-resistant lung cancer cells is accompanied by an increase in the characteristics of epithelial-mesenchymal transition and cancer stem cells, and the decrease in resistance to gefitinib may be due to its reduction in GRP78 [102]. It can be seen that GRP78 imparts stemness to cancer cells by transmitting signals, which participates in the resistance of cells to treatment.

## Clinical significance of GRP78

Since GRP78 is a key factor in multiple steps in tumor biology, GRP78 may have a significant clinical impact on lung cancer. First, GRP78 can be used as a target for tumor therapy. Secondly, the expression of UPR-related proteins, especially GRP78, can improve the therapeutic effect of drugs commonly used in the clinic. Finally, GRP78 can be used as a biomarker for the prognosis and evaluation of lung cancer patients.

### GRP78 can be used as a target for lung cancer treatment

Considering the importance of GRP78 in cancer cell survival, it is a prime target for anticancer drugs (Table 1). Interestingly, several naturally occurring compounds are thought to have anticancer activities that can inhibit the expression or activity of GRP78. Epigallocatechin gallate (EGCG) can bind to the ATPase domain of GRP78 and inhibit its catalytic activity [103]. In addition, several cytotoxins have been shown to inhibit GRP78 activity by direct lysis. For example, the bacterial AB5 subtilase cytotoxin (subAB) can specifically lyse GRP78 on a single amino acid, making it possible to use it for anticancer therapy [104]. In addition, the GRP78-targeted subtilase cytotoxin catalytic subunit is fused with epidermal growth factor (EGF-SubA) to make a variety of cancer cells sensitive to photodynamic therapy (PDT), including human squamous cell lung cancer SW-900. Hence, PDT and EGF-SubA can more effectively kill cancer cells when used in combination [97]. Versipelostatin inhibits the transcription of GRP78 target genes and initiates UPR-induced apoptosis under glucose deprivation [105, 106]. The macrocyclic compound also destroys certain components of the UPR and selectively kills glucose-deficient cancer cells. It can also inhibit tumor growth in xenografts when used in conjunction with cisplatin. GRP78 and related subtypes can be detected in the nucleus, mitochondria, cytoplasm, and ER of lung cancer cells [40, 107, 108]. GRP78 can also translocate to the cell surface [109], where it can act as a receptor for multiple signal transduction pathways, thereby amplifying its biological effects [110]. This provides an exciting opportunity to target cell surface GRP78 for developing new anticancer therapies. The kringle 5 domain (K5) of the human plasminogen can bind to GRP78 on the surface of endothelial cells and tumor cells and induce apoptosis [111, 112]. Targeted cell surface GRP78 antibodies slow the proliferation and colony formation of NSCLC cells and promote their apoptosis in vitro and in vivo. Yet, the antibody does not reduce the function of normal lung cells [113]. Tumor-targeting peptides are also promising drugs for tumor-targeting therapy. Several peptides have been clearly identified that bind to GRP78 on the surface of tumor cells [114–118], including lung cancer cells [119]. This finding can be used to target tumors and develop new targeted drugs for clinical and scientific research.

**Table 1:** GRP78-targeted drug therapies for cancer treatment

Drugs	Effects	Study
Versipelostatin	Inhibits transcription factors	Colon and stomach cancer [105]
EGCG	Binds to ATP binding domain and alters ATPase activity	Breast cancer [103]
Subtilase cytotoxin (AB5)	GRP78 proteolytic cleavage	Lung cancer [104]
GIRLRG	Targets cell surface GRP78	Lung cancer [119], glioma, and breast cancer [117]
CTVALPGGYVRVC		Melanoma [114]
WIFPWIQL		Prostate and breast cancer [116]
Bag-1 peptide		Prostate cancer [118]
Antibody		Lung cancer and glioblastoma [113]
Kringle 5 (K5)		Fibrosarcoma [111]

### ER stress and cancer cell sensitivity to therapy

Many research studies have indicated that the combination of existing chemotherapy drugs and UPR inhibitors or activators can prevents cell protection and induce cellular apoptosis, reinstate the sensitivity of cancer cells to chemotherapy drugs, and improve the efficacy of chemotherapy drugs with unclear molecular mechanisms. Inducing UPR signaling makes NSCLC cells sensitive to doxorubicin, at least partly through inactivation of the mTOR signaling pathway, which is mediated by eIF2α [120]. The targeted GRP78 antibody can inhibit the PIK3/AKT/mTOR signaling pathway and increase the radiosensitivity of NSCLC cells [113]. Currently, cisplatin is used in combination with other therapies in the clinic [121, 122]. Therefore, there is an urgent need to develop new strategies to reduce cisplatin-induced resistance in tumor cells while also reducing the common side effects of cisplatin-based treatment regimens [123]. The inhibition of glutamine fructose-6-phosphate aminotransferase (GFAT) activity downregulates the expression of GRP78 and activated IRE1α, a sensitive protein in response to unfolded proteins, thereby promoting cisplatin-induced apoptosis in NSCLC cells [124]. In addition, 4-PBA, which is an ER stress antagonist, enhances the sensitivity of human lung adenocarcinoma cells to DMAS [23]. However, the ER stress inducer THA sensitizes lung adenocarcinoma cells to icariin treatment [125]. Autophagy inhibitors significantly enhance the cytotoxicity of erlotinib to tyrosine kinase inhibitor (TKI)-resistant lung cancer cells through ER stress-induced apoptosis [126].

In view of the effects of oxidative stress on cell apoptosis and treatment, regulating oxidative stress and ER stress may be beneficial to the sensitivity of lung cancer to treatment. PDT produces high-cytotoxic ROS, especially singlet oxygen (^1^O_2_), which kills tumor cells through apoptosis/necrosis, while protecting normal tissues. However, the ER stress caused by PDT causes cells to initiate UPR to restore protein homeostasis and normalize ER function, and then cells can survive under this cytoprotective mechanism, which greatly reduces the efficacy of PDT [127]. Therefore, the combined use of drugs that target GRP78 destroys this protective mechanism, facilitates the accumulation of ROS in cells, and increases the sensitivity of lung cancer cells to PDT [97].

### GRP78 as a biomarker for early diagnosis, staging and prognosis of lung cancer

Due to the difficulties in diagnosing lung cancer, many patients present with advanced-stage disease at this initial assessment, which can lead to poorer prognoses [128–130]. Therefore, more information is needed to determine the prognosis of these patients. Molecular diagnostics can provide an objective and systematic classification of human cancers. However, researchers have been unable to identify any prognostic markers of lung cancer. GRP78 is highly enriched in the serum samples of patients with advanced-stage NSCLC. Compared with patients with lower GRP78 expression, patients with higher GRP78 expression have a shorter survival time (median overall survival, 39 months vs. 49 months). Hence, high GRP78 expression may indicate a shorter overall survival period [131]. Compared with the paired normal tissues of the same patient, the expression of genes and protein levels in cancer tissues was increased. In addition, GRP78 overexpression in cancer tissues is related to the degree and stage of tumor differentiation. GRP78 expression in poorly differentiated tumors is stronger than that in highly differentiated tumors, and the expression of stage III tumors is also stronger than that of stage I and II and tumors [7]. GRP78 can also be used as a prognostic indicator for predicting the response of patients to chemotherapy. For example, the rs430397 AA genotype in GRP78 is associated with reduced survival and higher recurrence in patients with advanced-stage NSCLC who receive platinum chemotherapy [132]. Therefore, GRP78 can be considered as a potential marker for evaluating the prognosis of lung cancer patients.

## Conclusion and outlook

The role of GRP78 in the growth, progression, and outcomes of lung cancer is becoming increasingly evident. GRP78 is the main regulatory protein of UPR, affecting both tumor cells and the tumor microenvironment. When changes in the microenvironment are felt, such as inflammation, hypoxia, nutritional deficiencies, and acidosis, GRP78 becomes overexpressed and migrates to the surface of tumor cells, leading to a series of cancer features.

Cell survival and death are heavily controlled by apoptosis and autophagy. The interaction between autophagy and apoptosis affects the stability of the intracellular environment, the clearance of dead cells, and clinical treatment. GRP78 has a dual role in the regulation of autophagy and apoptosis in lung cancer, and is highly dependent on the cell type and stimulation conditions. In some cases, autophagy has a protective effect on lung cancer cell apoptosis, while in other instances, autophagy and apoptosis have a synergistic effect. In general, mild ER stress can induce autophagy, promote the balance of the intracellular environment, and inhibit apoptosis. However, sustained and robust ER stress will induce apoptosis. Therefore, it is essential to know the factors that affect the balance between UPR-mediated survival and death responses. Since malignancies continually activate UPR in response to environmental stress, they may have also formed mechanisms to avoid UPR-induced apoptosis.

On the one hand, GRP78 overexpression in lung cancer cells may increase the secretion of GRP78, stimulate the formation of non-malignant stromal cells, and thereby support tumor growth. On the other hand, GRP78 overexpression induces EMT in lung cancer cells, contributing to the metastatic potential of lung cancer cells. Currently, there is a limited number of studies on the role of GRP78 in the tumor microenvironment. Therefore, it is necessary to conduct research in patient-derived cell culture models to further assess the role of GRP78, its involved UPR branch, and its downstream effects in the occurrence and progression of lung cancer. The increased GRP78 activity levels in lung cancer specimens may indicate that lung cancer cells may be able to overcome the harsh tumor microenvironment through adaptive mechanisms.

Since GRP78 may be the main factor in the occurrence of lung cancer, GRP78 can be used as a biomarker for the early diagnosis of lung cancer, or combined with existing biomarkers to improve the specificity and sensitivity of the lung cancer diagnosis. At present, some studies have shown that GRP78 can be used as a biomarker to evaluate the prognosis of lung cancer patients. Hence, the expression of GRP78 in the sera samples of lung cancer patients may be used to evaluate the response to treatment and predict the prognosis of lung cancer patients in the clinic. This may improve the accuracy of evaluation and treatment. In addition, lung cancer often resists existing treatment methods, leading to the enrichment of drug-resistant tumor cell subgroups, cancer recurrence, and ultimately patient death. GRP78 may be the primary factor for the development of drug resistance in lung cancer, as its specific inhibitors and targeted agents have excellent therapeutic prospects. Furthermore, GRP78-mediated ER stress can enhance the sensitivity of lung cancer cells to chemotherapy, so combined GRP78-based therapies should also be considered. Accurate cancer imaging is essential for the evaluation of patients before, during, and after treatment. Using this biomarker, the development of targeted imaging for lung cancer can be considered to specifically detect all cancer cells within a heterogeneous tumor while keeping normal cells intact. Since GRP78 plays a broad role in inflammation, novel GRP78-related immunotherapies could be developed in future.

In addition, how does GRP78 regulate lung cancer in multiple intracellular locations? Does GRP78 play a regulatory role in lung cancer-specific metastasis? What is the relationship between GRP78 and lung cancer metabolism? We recommend the establishment of a specific conditional knockout model of GRP78 in different parts of the cell and a model of lung cancer-specific metastasis, which will help to further reveal the function and regulatory effects of GRP78 in lung cancer. Our increased knowledge of these mechanisms may aid in the discovery of other tumor cell weaknesses, which can be translated into the development of novel treatment strategies in the future.

## Data Availability

Not applicable.

## Abbreviations

GRP78: Glucose-regulating protein 78
ER: Endoplasmic reticulum
UPR: Unfolded protein response
BiP: Binding protein
IRE1: Inositol-requiring enzyme 1
PERK: PRKR-like ER kinase
ATF6: Activating transcription factor 6
RNase: Ribonuclease
XBP1: X-box binding protein
ERAD: ER-related protein degradation
JNK: C-jun n-terminal kinase
TNF: Tumor necrosis factor
TRAF2: Tumor necrosis factor receptor-related factor 2
eIF2: Eukaryotic translation initiation factor 2a
ATF4: Activating transcription factor 4
CHOP: CCAAT/enhancer-binding protein-homologous protein
NRF2: Nuclear factor erythroid 2-related factor 2
SiP1: Site-1-protease
SiP2: Site-2-protease
bZIP: Basic leucine zipper
ROS: Reactive oxygen species
COPD: Chronic obstructive pulmonary disease
DMAS: β,β-Dimethylacrylshikonin
HOCl: Hypochlorous acid
EMT: Epithelial-mesenchymal transition
CAFs: Cancer-associated fibroblasts
GALNT6: Polypeptide *N*-acetylgalactosaminyltransferase-6
PDT: Photodynamic therapy
GFAT: Glutamine fructose-6-phosphate aminotransferase
TKI: Tyrosine kinase inhibitor
